# The Detection of Peripheral Neuropathy by Clinical Scales in Patients Diagnosed With Alcohol Use Disorder

**DOI:** 10.7759/cureus.70941

**Published:** 2024-10-06

**Authors:** Michail Papantoniou, Michail Rentzos, Evangelos Anagnostou, Elias Tzavellas, Thomas Paparrigopoulos, Panagiotis Kokotis

**Affiliations:** 1 Laboratory of Clinical Neurophysiology, First Department of Neurology, National and Kapodistrian University of Athens, Aeginition Hospital, Athens, GRC; 2 First Department of Neurology, National and Kapodistrian University of Athens, Aeginition Hospital, Athens, GRC; 3 First Department of Psychiatry, National and Kapodistrian University of Athens, Aeginition Hospital, Athens, GRC; 4 Laboratory Of Clinical Neurophysiology, First Department Of Neurology, National And Kapodistrian University Of Athens, Athens, GRC

**Keywords:** alcohol, clinical scales, large fiber, neuropathy, small fiber

## Abstract

Introduction

Peripheral neuropathy is a well-known manifestation of alcohol overconsumption, but neurophysiological confirmation of peripheral nerve damage is costly and sometimes involves invasive procedures. The aim of this study was to investigate the ability of commonly used clinical scales to detect the presence of neuropathy in patients with alcohol use disorder (AUD).

Methods

Data were collected retrospectively on 116 patients diagnosed with AUD and treated voluntarily in a detoxification special unit. Ninety-eight age and gender-matched healthy subjects without a diagnosis of AUD were used as the control group. The five tested clinical neuropathy scales were the Neuropathy Symptoms Score (NSS), the Neuropathy Disability Score, the Neuropathy Impairment Score (NIS), the Neuropathy Impairment Score of the Lower Limbs, and the modified Toronto Clinical Neuropathy Scale.

Results

The mean values of all tested clinical scales of the patients with AUD were significantly higher than the control group. All examined clinical scales were determined to be useful in discriminating between patients with neuropathy from patients without neuropathy. The strongest discrimination was seen with the NIS, including the best sensitivity and specificity for the range of scores obtained. All scales, except NSS, showed a stronger correlation with measures of large (LFN) than small fiber neuropathy (SFN).

Conclusion

Our study suggests that clinical scales could be used to detect peripheral neuropathy in patients with AUD when neurophysiological testing is not available. Moreover, we suggest that the NIS and the NSS scales would be most helpful in assessing LFN and SFN, respectively, in patients with AUD.

## Introduction

Alcohol use disorder (AUD) is well known to cause damage to the peripheral nervous system, involving both large and small fibers [1, 2]. Several neurophysiological tests have been used for the diagnosis of neuropathy in patients diagnosed with AUD, including nerve conduction studies (NCS), sympathetic skin response (SSR), quantitative sensory testing (QST), threshold techniques, and skin biopsies for the measurement of the intraepidermal nerve fiber density (IENFD) [1-12]. Nevertheless, conducting these procedures leads to high expenses and is often considered painful by the patients. There is a lack of a clinical tool for detecting and assessing alcohol-related peripheral neuropathy that is sensitive enough to detect early neuropathies and that correlates to measures of large (LFN) and small fiber neuropathy (SFN), which would be a great advantage to a targeted clinical examination and a symptom's questionnaire. There are multiple clinical neuropathy scales available, but most of them are commonly used for the detection of peripheral neuropathy in diabetic patients [13, 14]. In our previous study [7], we used the appropriate neurophysiological tests for the evaluation of large and small nerve fibers (NCS, QST, and SSR) and quantified the clinical findings and symptoms of our subjects by using two neuropathy scales: the Neuropathy Symptoms Score (NSS) questionnaire and the Neuropathy Disability Score (NDS) clinical examination tool. These scales were applied to our subjects in the form that was described and proposed by Dyck [15] for assessing diabetic patients, and it was hypothesized that clinical neuropathy scales would correlate with the patients diagnosed with AUD and peripheral neuropathy [7].

The aim of this study was to correlate neurophysiological findings with clinical neuropathy scales that do not involve neurophysiological testing in their batteries and to determine the ability of these scales to detect the presence of neuropathy in patients diagnosed with AUD.

## Materials and methods

Subjects

In this retrospective study, data were collected from our two recent studies [10-12], including 116 patients diagnosed with AUD who were admitted to the Detoxification Special Unit of Athens University Psychiatric Clinic on an inpatient basis from September 2017 to October 2022. Every participant (patients and healthy subjects) gave their informed written consent for the study, which was held in accordance with the Helsinki Declaration of 1975, as revised in Hong Kong in 1983. The study was approved by the Scientific Committee of Aeginition Hospital and the Bioethics Committee of the School of Medicine, National and Kapodistrian University of Athens. The subjects recruited for this study had to fulfill the DSM-5 diagnostic criteria for alcohol use disorder (a medical condition characterized by an impaired ability to stop or control alcohol use despite adverse social, occupational, or health consequences) [16] and be in age between 18 and 75 years [10-12]. Exclusion criteria for this study were: 1) peripheral neuropathy of any other known cause (metabolic, hereditary, infectious, etc.); 2) renal failure, dysthyroidism, hepatitis C virus, diabetes mellitus, anemia (of any cause), and human immunodeficiency virus; 3) medication with neurotoxic effects; and 4) allergy to lidocaine [10-12]. Ninety-eight age and gender-matched healthy subjects without a diagnosis of AUD were used as the control group.

Clinical neuropathy scales

Each participant (patients and healthy subjects) was assessed by peripheral nerve clinical evaluation by the same experienced physician (clinical neurologist), blinded to the results of the neurophysiological testing, using a validated version of the NSS [15], the NDS [15], the Neuropathy Impairment Score (NIS) [17, 18], the Neuropathy Impairment Score of the Lower Limb (NIS-LL) [19], and the modified Toronto Clinical Neuropathy Scale (mTCNS) [20], in the language of the group. The NSS scale refers to a symptom questionnaire, including symptoms of muscle weakness, sensory deficits, and autonomic dysfunction. The NDS, NIS, NIS-LL, and mTCNS scales are clinical examination tools, including grading from cranial nerves (NDS and NIS), muscle strength (NDS, NIS, and NIS-LL), superficial and deep sensation (NDS, NIS, NIS-LL, and mTCNS), and tendon reflexes (NDS, NIS, NIS-LL, and mTCNS). The NDS and NIS-LL scales are weighted towards motor deficits, whilst the NIS and mTCNS scales are weighted more towards sensory deficits. The scales above were selected according to the available data from our previous studies and the inclusion criterion of clinical neuropathy scales, which stated that neurophysiological testing was not included in their batteries.

Diagnosis of peripheral neuropathy

Neurophysiological tests were performed on subjects fulfilling the inclusion criteria described above for confirming the diagnosis of peripheral neuropathy, and included NCS, SSR, QST (heat detection threshold (HDT), cold detection threshold (CDT), heat pain threshold (HPT), and cold pain threshold (CPT)), and skin biopsies, diameter of 3 mm, 10 cm above the lateral malleolus of the leg [calculation of the average intraepidermal nerve fiber density (IENFD) per millimeter of epidermal length]. The methodology of neurophysiological testing and skin biopsy is described extensively in our previous studies [10-12]. NCS, QST, and SSR were performed in all subjects by a clinical neurophysiologist, while another clinical neurophysiologist performed skin biopsies and calculated the IENFD, both blinded to the results of clinical examination and symptoms interview.

Two abnormal laboratory tests (SSR and/or QST and/or IENFD) or the presence of at least one of the following symptoms: pain, postural hypotension, impotence, night diarrhea, and sphincter loss, and at least one abnormal laboratory test were required for the diagnosis of small fiber neuropathy [7, 10, 12]. Abnormal NCS and the presence of at least one of the following clinical signs: depressed or absent Achilles tendon reflexes, superficial and/or deep sensory deficit were required for the diagnosis of large fiber neuropathy [7, 10, 12].

Statistical analysis

Statistical analyses were performed using the SPSS Statistics 26 software package (IBM Inc., Armonk, New York). The sensitivity and specificity of the clinical scales were estimated by the area under the receiver operating characteristic (ROC) curve using the trapezoidal rule, and optimal cut-off values were calculated. We used the Shapiro-Wilk analysis to test the normality of our samples. Pearson's test was used to assess the statistical linear correlation of discrete variables with normal distribution, whilst Spearman's test was used for parameters with abnormal distribution. A probability level of 0.05 was chosen for statistical significance.

## Results

In this study, 73 male and 43 female patients with AUD were enrolled, with a median age of 50.4 ± 8.85 years (range 27-74). Peripheral neuropathy was diagnosed in 70 subjects (60.3%). Among them, pure LFN was found in 20 subjects (28.6%), pure SFN in 20 subjects (28.6%), and both large and small fiber neuropathy (LSFN) was diagnosed in 30 subjects (42.8%). Among subjects with LFN, 31 subjects had axonal sensory neuropathy, whilst 19 had axonal sensorimotor neuropathy. Among subjects with SFN, 28 subjects had abnormal or absent SSR, 40 had abnormal QST results, and 16 had IENFD values below the lower limit of normal values for their age. In the control group, there were 61 men and 37 women, with a mean age of 49.62 ± 3.21 years (range 25-75),

The mean values of all tested clinical neuropathy scales of the patients diagnosed with AUD were significantly higher than the age and gender-matched control group (NSS: mean 1.22 ± 1.09 vs. 0.06 ± 0.23; NDS: mean 2.63 ± 2.57 vs. 0.04 ± 0.21; NIS: mean 4.23 ± 3.87 vs. 0.05 ± 0.16; NIS-LL: mean 0.04 ± 0.21 vs. 95.62 ± 8.39; mTCNS: mean 3.03 ± 3.01 vs. 0.05 ± 0.16, respectively, Student's t-test p<0.001).

**Figure 1:**
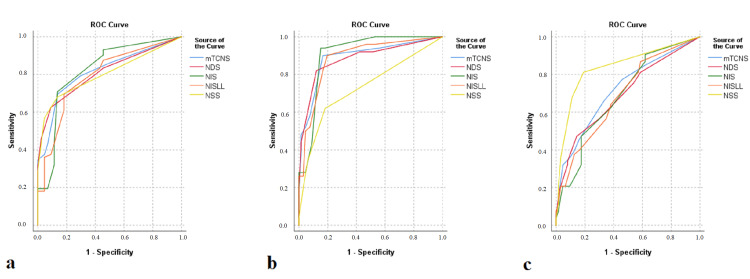
ROC analysis reveals values of NIS and NSS clinical scales and corresponding sensitivity and specificity for all types neuropathy (a), LFN (b), SFN (c) detection, in patients with alcohol use disorder ROC - receiver operating characteristic; NIS - Neuropathy Impairment Score; NSS - Neuropathy Symptoms Score; LFN - large fiber neuropathy; SFN - small fiber neuropathy

**Figure 2:**
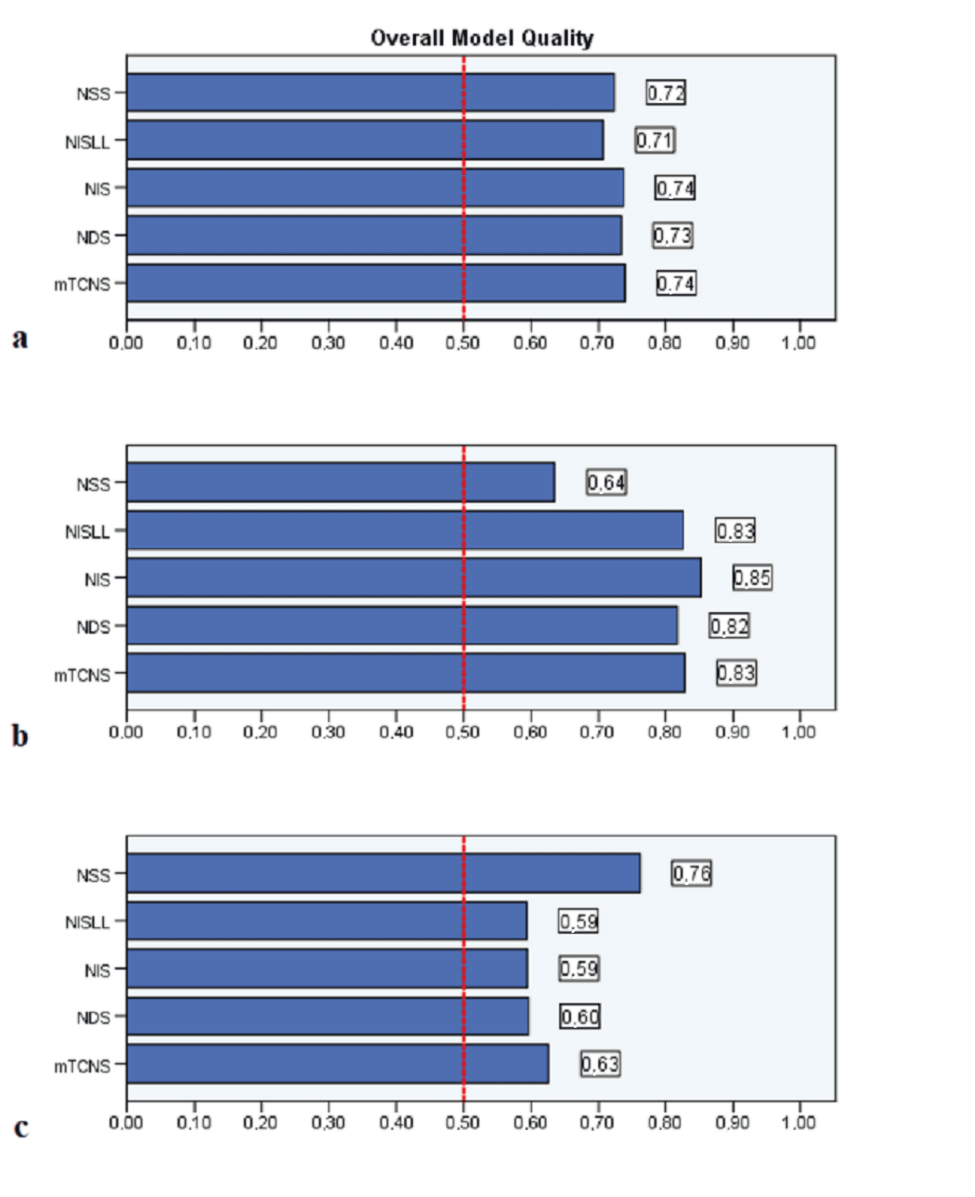
Overall model quality analysis for all types neuropathy (a), LFN (b), SFN (c) detection in patients diagnosed with alcohol use disorder LFN - large fiber neuropathy; SFN - small fiber neuropathy

Regarding the assessment of the scores on various clinical neuropathy scales in patients with and without alcohol-related peripheral neuropathy, the ROC sensitivity/specificity analysis indicated that the NIS and the mTCNS showed the greatest sensitivity and specificity among all of the examined clinical scales for detecting patients with neuropathy from those without neuropathy. All scales, except NSS, showed a stronger correlation with measures of LFN than SFN. The NIS scale performed best in detecting patients with LFN, whilst the NSS scale performed best in detecting patients with SFN (Figure 1). The cut-offs, as well as the sensitivity/specificity rates for these scales, are presented in Table 1. The areas under the ROC for patients with any kind of neuropathy, as well as for patients with either LFN or SFN, are compared in Table 2. All clinical scales tested showed very good accuracy in discriminating between patients with AUD, with and without neuropathy, and the control group (Figure 2).

**Table 1:** Cut-offs and determination of sensitivity/specificity for best performed scales in detecting neuropathy NIS - Neuropathy Impairment Score; mTCNS - modified Toronto Clinical Neuropathy Scale, NSS - Neuropathy Symptoms Score

Coordinates ROC Curve	Scale	Cut-off	Sensitivity	1-Specificity
Neuropathy type				
Neuropathy (all types)	NIS	4	0.708	0.136
Neuropathy (all types)	mTCNS	3	0.694	0.136
Large Fiber Neuropathy	NIS	4	0.940	0.152
Small Fiber Neuropathy	NSS	1	0.811	0.190

**Table 2:** Comparison between ROC for all neuropathy types and clinical scales ROC - receiver operating characteristic; NIS - Neuropathy Impairment Score; NSS - Neuropathy Symptoms Score; NDS - Neuropathy Disability Score; NIS-LL - neuropathy impairment score of the lower limbs; mTCNS - modified Toronto Clinical Neuropathy Scale

Neuropathy type	Scale	Area	Std. error	Sig.	Lower limit 95% CI	Upper limit 95% CI
Neuropathy (all types)	NIS	0.821	0.042	<0.001	0.738	0.904
	NSS	0.802	0.04	<0.001	0.723	0.881
	NDS	0.81	0.039	<0.001	0.734	0.887
	NIS-LL	0.792	0.043	<0.001	0.707	0.876
	mTCNS	0.816	0.039	<0.001	0.739	0.893
Large fiber neuropathy	NIS	0.908	0.028	<0.001	0.853	0.963
	NSS	0.731	0.049	<0.001	0.635	0.827
	NDS	0.884	0.034	<0.001	0.817	0.951
	NIS-LL	0.888	0.032	<0.001	0.826	0.95
	mTCNS	0.892	0.032	<0.001	0.828	0.956
Small fiber neuropathy	NIS	0.69	0.049	<0.001	0.594	0.786
	NSS	0.839	0.039	<0.001	0.762	0.916
	NDS	0.693	0.05	<0.001	0.596	0.791
	NIS-LL	0.689	0.049	<0.001	0.594	0.785
	mTCNS	0.72	0.048	<0.001	0.626	0.814

The scores of all tested scales were negatively correlated with the sural sensory nerve action potential (SNAP) amplitudes using linear regression. We also found that the scores of NIS and NSS were positively correlated with the CDT and the HDT results, respectively, while the scores of these two scales were negatively correlated with the IEFND results (Table 3).

**Table 3:** Correlation of clinical scales, sural SNAP amplitude, foot HDT, foot CDT and IENFD using linear regression SNAP - sensory nerve action potential; HDT - heat detection threshold; CDT - cold detection threshold; IENFD - intraepidermal nerve fiber density; NIS - Neuropathy Impairment Score; NSS - Neuropathy Symptoms Score; NDS - Neuropathy Disability Score; NIS-LL - neuropathy impairment score of the lower limbs; mTCNS - modified Toronto Clinical Neuropathy Scale

	Sural SNAP	HDT foot	CDT foot	IENFD
NIS	R2 =0.234, p<0.001	R2 =0.082, p=0.164	R2 =0.144, p=0.036	R2 =0.229, p=0.013
NSS	R2 =0.178, p<0.001	R2 =0.168, p=0.040	R2 =0.068, p=0.141	R2 =0.153, p=0.048
NDS	R2 =0.243, p<0.001	R2 =0.056, p=0.213	R2 =0.084, p=0.098	R2 =0.046, p=0.291
NIS-LL	R2 =0.239, p<0.001	R2 =0.018, p=0.412	R2 =0.042, p=0.187	R2 =0.014, p=0.570
mTCNS	R2 =0.265, p<0.001	R2 =0.022, p=0.440	R2 =0.108, p=0.078	R2 =0.002, p=0.822

## Discussion

In our recent studies [10-12], we examined several aspects of peripheral neuropathy in patients with AUD by using neurophysiological and objective structural tests. We also included the clinical neuropathy scales, NIS and NSS, as the most commonly used batteries in our everyday clinical practice for assessing peripheral neuropathy. These two scales were validated with QST, IENFD, and NCS, showing a significant association between NSS and QST, as well as between NIS and NCS, whilst both NSS and NIS had a significant association with IENFD, using linear regression, which was also confirmed by this study. In another study by our laboratory, where we examined large and small nerve fiber involvement in chronic alcohol-dependent subjects [7], the NDS scale was used instead of the NIS, showing a significant association between NDS and LFN. To our knowledge, there are no other studies using clinical neuropathy scales for assessing LFN and/or SFN in patients diagnosed with AUD.

In this retrospective study, we used the data from our two recent studies [10, 12] to expand the sample size (26 or 90 vs. 116 patients with AUD) and the number of tested clinical neuropathy scales (NSS and NIS vs. NSS, NDS, NIS, NIS-LL, and mTCNS), respectively, so that our results would be confirmed and better validated. We compared five commonly used clinical scales in diabetic neuropathy that do not involve neurophysiological testing in their batteries and aimed to determine the ability of these scales to detect the presence of neuropathy in patients diagnosed with AUD. We found that all five of the examined clinical neuropathy scales performed well and were able to distinguish subjects with alcohol-related peripheral neuropathy from those without, with a high degree of sensitivity and specificity. However, the NIS, followed by the mTCNS, had the greatest sensitivity and specificity for neuropathy (of any type), as well as the LFN subgroups. This may be because the NIS and the mTCNS scales are two grading scales that include a more detailed clinical examination regarding sensory grading (touch pressure, pin-prick, vibration, and joint position) and were used in this study for assessing a sensory-prominent length-dependent neuropathy, such as alcohol-related peripheral neuropathy. The NSS had the greatest sensitivity and specificity for the SFN subgroup, which was a reasonable finding since it is abstracted from a patient’s neurological history, including only symptom grading, and the diagnostic criteria for SFN required the presence of at least one symptom.

A limitation of our study was the relatively small number of subjects that were included because of the coexistence of other medical conditions, which had to be excluded, in the population of patients with AUD that were treated in the specialized detoxification unit. Another limitation of our study was that skin biopsy was not performed in all subjects that were included, so SFN might have remained undetected in some of these subjects [10-12].

## Conclusions

In this study, we tested five clinical neuropathy scales (NSS, NDS, NIS, NIS-LL, and mTCNS) and investigated their ability to detect alcohol-related neuropathy. Our findings suggest that all tested clinical scales performed well and could identify peripheral neuropathy in patients diagnosed with AUD, and could also be used in everyday clinical practice when essential neurophysiological testing is not available. We also suggest that the NIS could be most helpful in assessing LFN and the NSS in assessing SFN in these patients.
